# Metabolic Reprogramming in Oral Cancer: A Narrative Review of Therapeutic Perspectives with Emphasis on Dichloroacetate

**DOI:** 10.3390/cimb48070724

**Published:** 2026-07-16

**Authors:** Sara Senlle, Cécile Nicole, Patrícia M. A. Silva, Odília Queirós, Andrea Cunha

**Affiliations:** 1 UNIPRO-Oral Pathology and Rehabilitation Research Unit, University Institute of Health Sciences (IUCS)-CESPU, 4585-116 Gandra, Portugal; sarasenlle1791@hotmail.com (S.S.); cecilenicole123@gmail.com (C.N.); patricia.silva@cespu.pt (P.M.A.S.); andrea.cunha@iucs.cespu.pt (A.C.); 2 Associate Laboratory i4HB-Institute for Health and Bioeconomy, University Institute of Health Sciences (IUCS)-CESPU, 4585-116 Gandra, Portugal; 3 UCIBIO-Applied Molecular Biosciences Unit, Translational Toxicology Research Laboratory, University Institute of Health Sciences (1H-TOXRUN, IUCS)-CESPU, 4585-116 Gandra, Portugal

**Keywords:** oral cancer, Warburg effect, tumor metabolism, dichloroacetate, drug resistance

## Abstract

Oral squamous cell carcinoma (OSCC) represents a significant global health challenge characterized by high morbidity and mortality, frequently driven by therapeutic resistance and tumor aggressiveness. Metabolic reprogramming has emerged as a hallmark of OSCC, enabling tumor cells to sustain proliferation, survive under adverse microenvironmental conditions, and evade therapeutic stress. Recent advances in cancer metabolism have identified metabolic plasticity as a central determinant of OSCC progression and treatment failure, highlighting the need to integrate evidence on metabolic vulnerabilities and therapeutic opportunities. This narrative review aims to provide an updated overview of metabolic reprogramming in OSCC, with particular emphasis on the interplay between glycolysis, mitochondrial metabolism, glutamine metabolism, and fatty acid oxidation, and to discuss how these interconnected pathways may be therapeutically exploited. Although OSCC cells exhibit enhanced aerobic glycolysis, mitochondria remain functionally active and play critical roles in energy production, redox homeostasis, and metabolic adaptation. The therapeutic potential of targeting tumor metabolism is discussed, highlighting dichloroacetate (DCA) as a promising metabolic modulator capable of inhibiting pyruvate dehydrogenase kinase (PDK), restoring mitochondrial glucose oxidation, and partially reversing the glycolytic phenotype. The review also examines the current translational limitations of DCA, including toxicity, pharmacokinetic constraints, and compensatory metabolic adaptations that restrict its efficacy as a standalone therapy. Furthermore, potential synergistic strategies are explored, particularly the combination of DCA with paclitaxel, which enhances therapeutic efficacy through concurrent disruption of cytoskeletal integrity and metabolic homeostasis, thereby increasing cellular susceptibility to apoptosis and overcoming chemoresistance.

## 1. Introduction

Oral squamous cell carcinoma (OSCC), the most prevalent subtype of oral cancer, remains a major global public health concern, characterized by high morbidity and mortality despite advances in early detection and multimodal therapy [1]. Prognosis is particularly poor in advanced stages, where conventional treatments often fail to achieve durable responses. Among the biological features that drive tumor aggressiveness, altered cellular metabolism has emerged as a central hallmark of cancer, influencing tumor initiation, progression, and resistance to therapy [2,3,4]. A deeper understanding of the metabolic reprogramming that sustains oral cancer, particularly glycolytic rewiring and mitochondrial regulation, is therefore essential for identifying innovative therapeutic strategies capable of improving clinical outcomes [5].

One of the earliest recognized and most extensively studied metabolic alterations in cancer cells, particularly relevant to OSCC, is the Warburg effect, a phenomenon in which tumor cells preferentially rely on glycolysis for adenosine triphosphate (ATP) production even under normoxic conditions [4]. This metabolic shift from oxidative phosphorylation (OXPHOS) to aerobic glycolysis leads to increased glucose uptake and elevated lactate production [3,6,7]. The consequent acidification is a well-established driver of tumor aggressiveness, promoting invasion, metastasis, immune evasion, and resistance to standard therapies [7,8]. In OSCC, these metabolic adaptations support rapid cellular proliferation while contributing to treatment resistance, highlighting the therapeutic potential of targeting glycolytic dependence as a strategy to overcome metabolic vulnerabilities in cancer cells [9].

Given the limitations of current treatment modalities, particularly in advanced or recurrent OSCC, there is growing interest in metabolism-targeted therapeutic strategies that disrupt key pathways involved in glycolysis, lactate transport, and mitochondrial regulation [5,6]. Glycolytic inhibitors and metabolic modulators have emerged as promising candidates, either as standalone agents or as adjuvants to conventional therapies [10]. By interfering with metabolic plasticity, a key driver of tumor adaptation, survival, and chemoresistance, these strategies may enhance therapeutic efficacy and overcome resistance mechanisms [9].

This review integrates current knowledge on metabolic plasticity in OSCC, emphasizing the interplay between glycolysis, mitochondrial metabolism, glutaminolysis, and fatty acid oxidation. Furthermore, it critically discusses the translational limitations of dichloroacetate (DCA) and highlights the rationale for biomarker-driven and combination-based metabolic therapies as future therapeutic strategies in OSCC.

## 2. Material and Methods

This work was conducted as a narrative review to provide a comprehensive overview of metabolic reprogramming in OSCC, with particular emphasis on the therapeutic potential of DCA and emerging metabolism-targeted therapeutic strategies. The literature search was performed using the PubMed database. Search terms included combinations of OSCC, Warburg effect, tumor metabolism, glycolysis, DCA, pyruvate dehydrogenase kinase (PDK), glutaminolysis, fatty acid oxidation (FAO), and drug resistance. Publications in English available up to June 2026 were considered. To provide contemporary perspectives, seminal studies describing fundamental concepts in cancer metabolism, including Otto Warburg’s original work, were considered alongside recent publications reflecting current advances in the field. The review included peer-reviewed original research articles, review articles, clinical studies, and clinical trial reports relevant to the metabolic biology of OSCC and metabolism-targeted therapeutic approaches. Studies were selected based on their scientific relevance to the objectives of the review, with particular attention to evidence addressing glycolytic reprogramming, mitochondrial plasticity, glutaminolysis, FAO, and the therapeutic opportunities and translational limitations of DCA. This approach enabled an integrated discussion of the metabolic adaptability of OSCC and its implications for the development of biomarker-driven and combination-based therapeutic strategies.

## 3. Tumor Cell Metabolism and the Glycolytic Phenotype

Although cancers arise from diverse tissues and exhibit substantial genetic and phenotypic variability, tumor cells share a set of fundamental biological capabilities, including uncontrolled proliferation, evasion of apoptosis, and the ability to invade and metastasize [3]. The Hallmarks of Cancer framework offers a widely accepted model for explaining the biological trait that enable tumor initiation, progression, and dissemination [11]. The updated model recognizes metabolic reprogramming as a central feature of malignant progression, reflecting the ability of cancer cells to adapt their energy production and biosynthetic pathways to support growth, survival, and dissemination. Among these adaptations, the glycolytic phenotype represents a key metabolic hallmark associated with tumor aggressiveness and therapeutic resistance, aligning with the focus of this review.

Within this framework, genomic instability and tumor-promoting inflammation act as enabling characteristics that facilitate the acquisition of hallmark traits. Additional conceptual dimensions have recently been proposed, further extending the framework and the mechanisms through which malignant phenotypes emerge [11].

### 3.1. Metabolic Reprogramming in Oral Cancer: The Warburg Effect and Beyond

**Figure 1:**
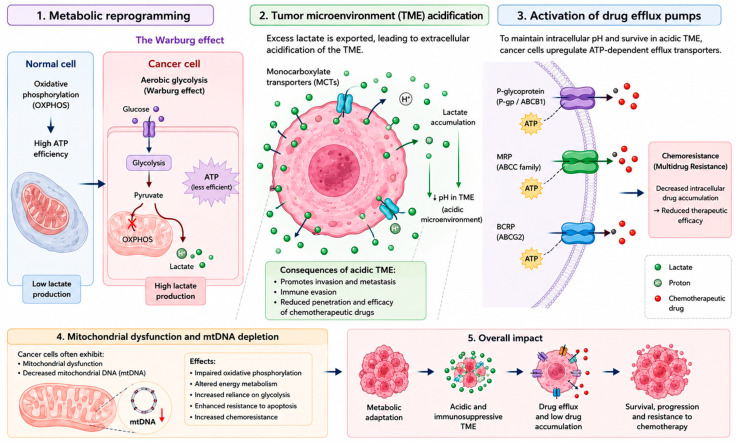
Metabolic reprogramming and chemoresistance in cancer cells. This figure illustrates key mechanisms underlying chemoresistance in the context of tumor metabolic reprogramming. Cancer cells preferentially adopt a glycolytic metabolic phenotype, a phenomenon known as the Warburg effect, characterized by enhanced glycolytic flux and increased lactate production even in the presence of sufficient oxygen availability (the red cross indicates inhibition of OXPHOS) (**1**). Lactate accumulation acidifies the tumor microenvironment, promoting tumor progression and reducing drug efficacy (**2**). To survive in these acidic conditions, cancer cells upregulate drug efflux pumps (such as P−glycoprotein), thereby decreasing intracellular drug accumulation (**3**). In addition, mitochondrial dysfunction and mtDNA depletion (indicated by the downward red arrow) enhance metabolic adaptation and resistance to apoptosis, ultimately contributing to tumor survival and chemotherapy resistance (**4**), with an overall increase in tumor survival, aggressiveness and chemoresistance (**5**). Black arrows indicate the direction of metabolic processes or molecular transport, whereas blue arrows indicate the progression between the sequential mechanisms illustrated. Created by the authors using Canva Pro.

Cancer cells undergo profound metabolic reprogramming to sustain uncontrolled proliferation, survival, and invasion. Among these alterations, the Warburg effect is one of the most extensively characterized metabolic hallmarks of cancer, including in OSCC. This metabolic adaptation confers a selective advantage by enabling tumor cells to thrive in hypoxic environments, maintain biosynthetic activity, and evade apoptosis. First described by Otto Warburg in the early 20th century, this phenomenon refers to the preferential use of glycolysis over OXPHOS for ATP generation, even under normoxic conditions [4,12]. In OSCC, maintenance of the glycolytic phenotype is driven by increased expression of metabolic enzymes, including hexokinase 2 (HK2), lactate dehydrogenase A (LDHA), and pyruvate kinase M2 (PKM2), which together promote enhanced glucose utilization and lactate generation [3,13]. These metabolic adaptations reshape the tumor microenvironment and contribute to immune evasion, chemoresistance, and metastatic progression (Figure 1).

Several interconnected metabolic pathways sustain the aggressive phenotype of oral cancer cells. At the glycolytic level, OSCC cells frequently overexpress glucose transporters GLUT1 and GLUT3, facilitating elevated glucose uptake [3,14]. Conversion of pyruvate to lactate via LDHA leads to accumulation of extracellular lactate and acidification of the tumor microenvironment. This acidic milieu promotes angiogenesis, immune suppression, and extracellular matrix degradation, enhancing invasion and metastasis [8,15]. Lactate accumulation further impairs cytotoxic immune cell function, reinforcing tumor-promoting mechanisms. Beyond aerobic glycolysis, mitochondrial metabolism also plays a relevant role: although glycolysis predominates, subsets of OSCC cells retain functional mitochondrial respiration, contributing to metabolic plasticity and resistance to therapy [8,16]. While aerobic glycolysis remains a dominant metabolic feature of OSCC, tumor cells rarely depend on a single metabolic pathway. Instead, they exploit a network of interconnected metabolic programs, including glutaminolysis, lipid metabolism, and FAO, which collectively contribute to metabolic adaptability and therapeutic resistance. Mutations in mitochondrial DNA (mtDNA) can impair electron transport chain activity, increase reactive oxygen species (ROS) production, and promote tumor progression. Rather than a complete shutdown, these alterations reflect a metabolic rewiring that supports redox balance and biosynthesis [17]. In parallel, glutaminolysis provides anaplerotic input to the tricarboxylic acid (TCA) cycle and supplies nitrogen for nucleotide and amino acid biosynthesis [18,19]. OSCC cells often exhibit elevated expression of glutaminase (GLS), reflecting a strong dependence on glutamine for survival and proliferation [20]. Lipid metabolism represents yet another dimension, along with FAO. Overexpression of fatty acid synthase (FASN) and stearoyl-CoA desaturase (SCD1) has been associated with tumor progression, invasion, and poor prognosis [21]. FAO provides an alternative energy source under hypoxic or nutrient-limited conditions, supporting tumor survival and contributing to therapeutic resistance [22]. Pharmacological inhibition of FAO, particularly through carnitine palmitoyltransferase 1 (CPT1) blockade with etomoxir, has demonstrated antitumor effects in preclinical cancer models by impairing NADPH production, increasing ROS accumulation, and inducing ATP depletion and cell death. However, the clinical development of etomoxir has been limited by concerns regarding off-target effects and hepatotoxicity [23].

Given the strong metabolic dependencies of OSCC, several therapeutic approaches are under investigation. Glycolysis inhibitors (e.g., 2-deoxyglucose (2-DG) and DCA) disrupt metabolic flux and promote mitochondrial reactivation, leading to apoptosis [24]. LDHA inhibitors such as FX11 have demonstrated antitumor activity in preclinical models [25]. GLS inhibitors such as CB-839 are currently under clinical investigation in solid tumors, including head and neck cancers [26]. FAO inhibitors such as etomoxir remain of interest in preclinical settings [14].

Metabolic regulation in OSCC is a dynamic and highly adaptable process that confers significant survival advantages to tumor cells [27]. Cancer cells exhibit pronounced metabolic plasticity, enabling continuous adaptation to environmental stress and therapeutic pressure [28]. Understanding these metabolic vulnerabilities is essential for the development of novel therapeutic approaches that interfere with energy metabolism and biosynthetic networks that sustain tumor growth [29]. Future research should prioritize combination treatments that simultaneously target multiple metabolic pathways, as this multi-layered approach may better overcome resistance mechanisms and improve clinical outcomes [9].

### 3.2. Mitochondrial Reprogramming and Oxidative Metabolism

Recent evidence indicates that, in OSCC, mitochondria are not functionally suppressed but instead undergo an adaptive metabolic reprogramming. These organelles retain significant bioenergetic and biosynthetic activity, contributing to oncogenic signaling, redox homeostasis, and metabolic plasticity [16]. Importantly, mitochondrial respiration is often modulated rather than abolished, allowing tumor cells to balance ATP production and ROS generation. This regulation prevents excessive ROS accumulation that could otherwise trigger mitochondrial-dependent apoptosis [30,31].

Mitochondrial dysfunction in OSCC frequently arises from mutations in mtDNA and deregulation of TCA cycle enzymes, including succinate dehydrogenase [32]. Notably, these alterations do not abolish oxidative metabolism; instead, they modify electron transport chain dynamics, leading to increased ROS production. Elevated ROS levels contribute to genomic instability, activation of retrograde signaling pathways, and promotion of tumor progression and invasive behavior [17,32].

This perspective aligns with the work of Tataranni et al., who emphasize that even in highly glycolytic tumors, mitochondria remain essential hubs for supplying carbon skeletons through the TCA cycle to support the biosynthesis of lipids, amino acids, and other macromolecules required for rapid proliferation [33]. Thus, OXPHOS does not disappear; instead, it coexists with aerobic glycolysis, reflecting the metabolic flexibility characteristic of OSCC cells. This dual bioenergetic capacity allows tumor cells to balance ATP production with the generation of biosynthetic precursors. Such coexistence of glycolysis and OXPHOS is increasingly recognized as a hallmark of metabolic plasticity in aggressive solid tumors. Mitochondrial reprogramming also contributes to apoptosis resistance, partly through modulation of mitochondrial membrane potential and the regulation of Bcl-2 family proteins, which may reduce the effectiveness of conventional therapies [17]. Furthermore, as highlighted by Wu et al., mitochondria function as a metabolic “safety net,” engaging alternative pathways such as the GABA shunt, a metabolic bypass that converts the neurotransmitter GABA into succinate to fuel the TCA cycle, to maintain energy flux and cellular integrity when the primary glycolytic routes are compromised [34,35]. Together, these findings underscore that mitochondria remain dynamic and indispensable components of OSCC metabolism. Their ability to sustain oxidative metabolism, buffer metabolic stress, and regulate cell survival pathways provides tumor cells with the metabolic robustness necessary for progression, therapeutic resistance, and adaptation to fluctuating microenvironmental conditions. Consequently, targeting mitochondrial signaling and oxidative metabolism, alone or in combination with glycolytic inhibition, offers a potential means of overcoming metabolic resilience in OSCC [36].

### 3.3. Metabolic Plasticity and Alternative Fuel Pathways

The metabolic phenotype of OSCC is shaped by a complex network of signaling pathways that confer tumor cell plasticity and enable rapid adaptation to fluctuating microenvironmental conditions. A central component of this regulatory system involves mitochondria-associated membranes (MAMs), specialized contact sites linking the endoplasmic reticulum and mitochondria that regulate calcium (Ca^2+^) transfer, lipid metabolism, and stress-response signaling [37,38].

In malignant cells, dysregulation of MAMs structure and function enhances metabolic flexibility, facilitating dynamic shifts between glycolysis and OXPHOS in response to hypoxia, nutrient deprivation, or therapeutic pressure [33]. Controlled Ca^2+^ flux into mitochondria is essential for the activation of TCA cycle dehydrogenases and sustaining oxidative metabolism. In contrast, disruption of Ca^2+^ homeostasis promotes a glycolytic phenotype, reduces mitochondrial apoptotic signaling, and enhances survival under stress.

Hypoxia-inducible factor 1-alpha (HIF-1α) also contributes to metabolic adaptation and epithelial–mesenchymal transition (EMT) through induction of transcription factors such as Snail and mesenchymal markers, including Vimentin [39,40,41].

Mitochondrial signaling further integrates metabolic adaptation through ROS and metabolite-driven pathways [17]. Thus, metabolic signaling in OSCC extends far beyond energy production: it functions as an integrated regulatory network governing tumor progression and resistance to therapy [5]. Recent studies highlight metabolic plasticity as a key mechanism of therapeutic escape. While Zong et al. emphasize the genomic instability and chemoresistant phenotypes driven by ROS-mediated signaling, Tubbs & Rieusset underscore the structural resilience of MAMs as a determinant of metabolic adaptability [17,37]. Together, these findings illustrate how OSCC cells can rewire metabolic fluxes to bypass targeted inhibitors when underlying signaling networks remain intact.

The ability of OSCC cells to dynamically reprogram their metabolism underscores the need for therapeutic strategies that target adaptive metabolic pathways rather than isolated metabolic nodes [17,37]. As long as mitochondrial signaling crosstalk remains functional, tumor cells may compensate for metabolic inhibition through activation of alternative metabolic pathways. Therefore, future multi-target approaches must account for this metabolic flexibility to effectively prevent therapeutic resistance and limit tumor progression [9].

## 4. Therapeutic Targeting of Metabolic Reprogramming in Oral Squamous Cell Carcinoma

As described in Section 3, OSCC cells frequently exhibit a glycolytic phenotype driven by HIF-1α signaling [12]. This metabolic shift is not merely a by-product of rapid proliferation but a central driver of tumorigenesis. In OSCC, glycolytic reprogramming is frequently intensified by stabilization of HIF-1α, a transcription factor activated under intratumoral hypoxia. As originally demonstrated by Semenza et al., hypoxic regions within solid tumors initiate a transcriptional program that upregulates glucose transporters such as GLUT1 and key rate-limiting enzymes, thereby reinforcing a high glycolytic flux [15].

Different studies highlight complementary roles for glycolysis in cancer. Heiden et al., emphasize that glycolysis supports rapid ATP generation required for proliferation [4]. In parallel, Hsu et al. argue that glycolysis functions as a biosynthetic hub, with intermediates diverted into pathways such as the pentose phosphate pathway (PPP) to produce NADPH and ribose-5-phosphate, essential for redox balance and nucleotide synthesis [32]. Additionally, Doherty et al. highlight the impact of lactate secretion on the tumor microenvironment, where extracellular acidification promotes invasion, matrix degradation, and immune suppression [8].

Targeting rate-limiting glycolytic enzymes has emerged as a promising therapeutic strategy, as many malignant cells, including OSCC, exhibit a strong dependence on aerobic glycolysis [4]. These enzymes (e.g., HK2 and LDHA) represent well-recognized metabolic vulnerabilities in OSCC, supporting the rationale for glycolysis-targeted therapeutic strategies [4,42]. Collectively, these effects reduce ATP availability and restrict the supply of biosynthetic intermediates [4]. In OSCC research, 2-DG remains a widely studied glycolytic inhibitor, acting as a glucose analog that competitively inhibits HK2 and induces energetic stress [43]. Compounds such as Lonidamine have also been explored for their ability to interfere with glycolytic enzymes and mitochondrial function, sensitizing tumor cells to apoptosis [44].

### Metabolic Plasticity as a Source of Therapeutic Vulnerabilities in Oral Squamous Cell Carcinoma

Despite the therapeutic potential of glycolytic inhibition, clinical efficacy is often limited by the remarkable metabolic plasticity of OSCC cells. Under metabolic stress, tumor cells can compensate by increasing their dependance on glutaminolysis and other alternative pathways, highlighting glutamine metabolism as an attractive therapeutic target [5]. When glycolysis is impaired, OSCC can activate alternative metabolic pathways, particularly glutaminolysis and fatty acid metabolism, to preserve energy production and biosynthetic capacity [22,26,45]. These compensatory pathways highlight the limitations of single-agent glycolytic inhibitors and underscore the need for multi-target metabolic strategies capable of disrupting the interconnected metabolic networks that sustain OSCC progression.

Several pharmacological strategies have been developed to exploit these metabolic vulnerabilities. Numerous inhibitors have been developed to target these key enzymes: CB-839 (telaglenastat), an allosteric inhibitor of GLS1 currently in phase I and II clinical trials; BPTES; V-9302 targeting ASCT2; and combinatorial approaches associating glutaminolysis inhibition with glycolysis blockade or immunotherapy [20,46,47]. Recent evidence has also highlighted the role of mitochondrial dynamics in regulating glutaminolysis and cancer stem cell properties in OSCC, suggesting novel therapeutic synergies [48].

Beyond its role in nucleotide and amino acid synthesis, glutamine serves as a precursor of mitochondrial citrate, which is subsequently used as a substrate for de novo lipogenesis through the enzymes ACLY and FASN, a process particularly relevant in the context of tumor hypoxia. Overexpression of Fatty Acid Synthase (FASN), a central enzyme in de novo lipogenesis, has been robustly documented in OSCC, with a direct correlation with cervical lymph node metastasis and overall patient survival [49]. In parallel, alterations in fatty acid profiles, sphingolipid homeostasis, and cholesterol accumulation in the tumor microenvironment have been identified as additional pro-tumorigenic mechanisms [50]. More recently, lipid droplets have been described as active pro-metastatic organelles acting via EMT and the PI3K/AKT/mTOR pathway [51]. The oncogene c-Myc, widely documented as overexpressed in OSCC, simultaneously coordinates glutamine uptake and utilization, through regulation of the transporter SLC1A5 and GLS, and the activation of de novo lipogenesis via FASN and ACLY [52,53]. This dual metabolic regulation promotes glutathione synthesis, reduces cisplatin-induced oxidative stress, and sustains lipid production required for cell proliferation and survival under adverse conditions [52,53,54].

FAO has emerged as an additional metabolic adaptation mechanism in OSCC. Recent evidence indicates that enhanced FAO may support tumor progression and metastatic dissemination by providing metabolic flexibility under conditions of energetic stress [55]. In particular, Pang et al. demonstrated that IRF2BP2 overexpression promotes lymph node metastasis in OSCC through Drp1-dependent mitochondrial fission, leading to increased CPT1A expression and FAO activity. Genetic silencing of IRF2BP2 reduced FAO, invasion, lymphovascular invasion, epithelial–mesenchymal transition and lymph node metastasis, whereas CPT1A overexpression partially rescued these effects, highlighting CPT1A-dependent FAO as a potential therapeutic vulnerability in metastatic OSCC [55,56]. Moreover, recent evidence suggests that FAO may function as a metabolic compensatory pathway when glutamine availability is limited. Cai et al. demonstrated that glutamine-deprived OSCC cells enhance lipid utilization through increased LDL uptake and activation of PPARα signaling, with FAO supplying substrates to the TCA cycle while sustaining cellular energy production. These adaptations enabled tumor cell survival despite impaired glutamine metabolism, illustrating the remarkable metabolic flexibility of OSCC cells [57]. Collectively, these findings demonstrate that OSCC cells exhibit remarkable metabolic flexibility, enabling them to switch between glycolysis, glutaminolysis, and FAO according to nutrient availability and therapeutic pressure. This adaptability may contribute to treatment failure by enabling tumor cells to maintain ATP production, redox homeostasis, and biosynthetic capacity despite inhibition of a single metabolic pathway. Consequently, therapeutic strategies targeting glycolysis alone are unlikely to achieve durable responses in all patients. Instead, combinatorial approaches simultaneously targeting multiple metabolic dependencies may be required to overcome compensatory adaptations and improve treatment efficacy in metabolically heterogeneous OSCC tumors.

Table 1 summarizes the main metabolism-targeted therapeutic strategies explored in OSCC.

## 5. DCA as a Potential Therapeutic Agent

DCA has emerged as a promising metabolic modulator in preclinical studies, capable of partially reversing the Warburg effect by promoting mitochondrial glucose oxidation. Its therapeutic rationale is based on the inhibition of PDK, resulting in decreased inhibitory phosphorylation of the pyruvate dehydrogenase (PDH) complex and enhanced conversion of pyruvate into acetyl-CoA, thereby promoting mitochondrial oxidative phosphorylation (OXPHOS) over aerobic glycolysis [24,33].

**Figure 2:**
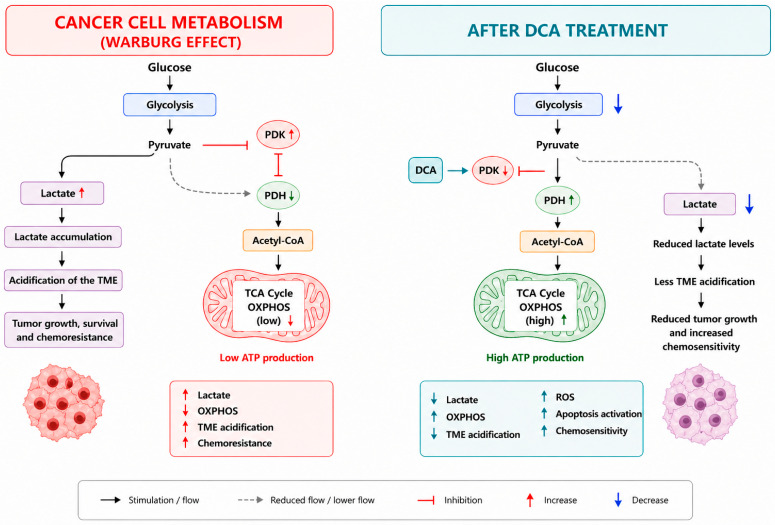
Dichloroacetate (DCA), partially reverses the Warburg effect and restores mitochondrial oxidative metabolism in oral squamous cell carcinoma (OSCC). This figure illustrates the metabolic reprogramming induced by DCA. In OSCC, enhanced glycolysis and suppression of mitochondrial oxidative phosphorylation (OXPHOS) promote the Warburg phenotype, whereby glucose is preferentially metabolized to lactate despite oxygen availability, leading to increased lactate production, accumulation of lactate, acidification of the tumor microenvironment (TME), and reduced mitochondrial oxidative phosphorylation (OXPHOS), resulting in low ATP production and promoting tumor progression, survival, and chemoresistance. Following treatment with DCA, inhibition of pyruvate dehydrogenase kinase (PDK), leads to reactivation of pyruvate dehydrogenase (PDH), facilitating the conversion of pyruvate into acetyl-CoA and restoring mitochondrial TCA cycle activity and OXPHOS. This metabolic shift reduces lactate production and tumor activity, thereby promoting apoptosis, suppressing OSCC tumor growth, and enhancing chemosensitivity. Created by the authors using Canva Pro.

In OSCC, several PDK isoforms, particularly PDK1 and PDK3, are frequently overexpressed, contributing to metabolic reprogramming characterized by sustained glycolysis and lactate production even under normoxic conditions. In this context, PDK inhibition by DCA has been proposed to restore PDH activity and redirect pyruvate toward mitochondrial oxidative metabolism [10] (Figure 2).

### 5.1. Preclinical Evidence in Oral Squamous Cell Carcinoma

The biomedical effects of PDK inhibition have translated into encouraging antitumor activity in preclinical models. This metabolic reprogramming has been associated in preclinical models with increased mitochondrial activity, ROS production, and destabilization of mitochondrial membrane potential, potentially promoting cytochrome c release and activation of intrinsic apoptotic pathways [17]. Importantly, however, the extent to which these metabolic effects translate into selective tumor cell death in clinically relevant settings remains uncertain. While DCA may preferentially affect highly glycolytic tumor cells, its therapeutic selectivity in heterogeneous tumor populations and in vivo human tumors is not fully established [10].

Preclinical studies using OSCC cell lines such as SCC-9 and CAL-27 have demonstrated dose-dependent reductions in cell viability following DCA exposure, typically in the millimolar range (10–50 mM), with associated changes in apoptotic markers, including decreased Bcl-2 expression and increased cleavage of pro-caspase-3, consistent with activation of apoptotic signaling pathways. Similar findings have been reported across other cancer models, where DCA has been shown to influence mitochondrial polarization and oxidative metabolism. However, it is important to note that these concentrations are substantially higher than plasma levels achievable in clinical settings (approximately 0.5–1 mM in therapeutic dosing), which raises concerns regarding pharmacological relevance and translational validity of many in vitro findings [58,59]. In vivo xenograft models, using doses of approximately 200–500 mg/kg/day, further corroborate these findings, showing significant tumor growth inhibition [17].

Beyond its direct metabolic effects, DCA has been associated with modulation of tumor microenvironment acidification and increased sensitivity to cytotoxic agents. Preclinical studies suggest that DCA may enhance the activity of conventional chemotherapeutic agents, paclitaxel (PTX), through metabolic reprogramming that increases oxidative stress and impairs energy-dependent resistance mechanisms [10,60]. While these combinatorial effects are mechanistically plausible, they remain largely restricted to preclinical models.

### 5.2. Challenges and Limitations of DCA-Based Anticancer Therapy

Despite these encouraging experimental findings, the clinical translation of DCA remains significantly constrained by toxicity concerns and pharmacokinetic limitations. The most frequently reported adverse effect is reversible peripheral neuropathy, which may occur in a dose-dependent manner and represents the principal factor limiting long-term administration [61]. Clinical manifestations include distal numbness, gait disturbances, and, in some cases, ataxia [62]. Proposed mechanisms involve impaired myelination resulting from DCA-mediated metabolic alterations in Schwann cells, which rely heavily on glycolytic metabolism [63]. Additional adverse effects reported in clinical studies include gastrointestinal symptoms such as nausea, vomiting, anorexia, and abdominal pain, as well as elevations in hepatic transaminases, highlighting the need for careful monitoring during prolonged treatment [62,63,64,65].

Although severe hepatotoxicity appears less common in humans than in animal models, chronic exposure may still pose safety concerns, particularly at higher doses [63]. Another important limitation relates to the pharmacological gap between the concentrations required to achieve robust antitumor effects in vitro and those safely attainable in patients. Most preclinical studies demonstrating marked reductions in tumor cell viability employ DCA concentrations in the millimolar range, whereas clinically achievable plasma levels are substantially lower [63]. Consequently, DCA is generally considered to exert predominantly cytostatic rather than directly cytotoxic effects at therapeutic doses [63]. Moreover, DCA inhibits its own metabolism through inactivation of glutathione transferase zeta 1 (GSTZ1), resulting in progressive drug accumulation and reduced clearance during repeated administration, which may further increase the risk of chronic toxicity [62,63].

Beyond toxicity and pharmacokinetic constraints, theoretical concerns have also been raised regarding the potential impact of DCA on antitumor immunity, since activated T cells and natural killer cells depend on glycolytic metabolism for optimal function [66,67]. Collectively, these factors may contribute to the discrepancy between the promising preclinical activity of DCA and the modest efficacy observed in clinical settings. Nevertheless, these limitations do not preclude its therapeutic use, but rather support the rationale for exploring DCA in combination-based approaches, where lower and potentially safer doses may enhance the efficacy of conventional anticancer therapies [63,68]. Furthermore, tumor cells can activate compensatory metabolic pathways that attenuate the effects of PDK inhibition. These include upregulation of the pentose phosphate pathway (PPP), enhanced antioxidant defense systems, and alternative anaplerotic routes such as the GABA shunt, all of which contribute to metabolic plasticity and may reduce susceptibility to DCA-induced metabolic stress [33,69]. Such adaptive mechanisms likely represent a key reason why DCA monotherapy has shown limited and inconsistent efficacy in translational and clinical contexts.

Taken together, current evidence suggests that DCA, while mechanistically well-founded as a PDK inhibitor and metabolic modulator, has limited therapeutic efficacy as a standalone agent in OSCC. Its greatest clinical potential may lie in rational combination strategies designed to exploit metabolic vulnerabilities while preventing compensatory adaptation. These may include co-targeting glycolysis, glutamine metabolism, or antioxidant defense systems to amplify metabolic stress and overcome tumor plasticity. However, these strategies require validation in clinically relevant preclinical models and well-designed translational studies before their therapeutic potential can be fully established [70,71].

## 6. Combination-Based Therapeutic Approaches and Clinical Translation

Growing evidence suggests that the clinical effectiveness of metabolism-targeted therapies in OSCC depends largely on their integration with standard therapeutic modalities. Recent evidence highlights a potent synergy between glycolytic inhibitors and platinum-based chemotherapy [17]. By reducing ATP availability and disrupting the acidic microenvironment generated by lactate secretion, agents such as 2-DG and DCA have been shown to enhance the cytotoxic effects of DNA-damaging compounds, like cisplatin, in preclinical cancer models [24,72]. This combined approach weakens the metabolic defenses that underlie chemoresistance in advanced oral tumors, thereby enhancing therapeutic efficacy [9].

### 6.1. Metabolic Plasticity and Immunometabolic Strategies

The interplay between tumor metabolism and immunotherapy has also emerged as a critical area of investigation. As described by Doherty et al., the high glycolytic flux characteristic of OSCC leads to substantial extracellular lactate accumulation, acidifying the tumor microenvironment [8]. This low pH suppresses the activity of cytotoxic T cells and natural killer (NK) cells, effectively creating an immunosuppressive niche [9]. Metabolic inhibitors that neutralize this acidity may therefore restore immune cell function and improve the performance of immune checkpoint inhibitors such as anti-PD-1/PD-L1 agents, particularly in aggressive or treatment-refractory carcinomas [73].

Although glucose metabolism is a primary therapeutic target, OSCC cells exhibit remarkable metabolic flexibility. As demonstrated by Gross et al., glutaminolysis serves as a crucial compensatory pathway that sustains cell survival when glycolysis is inhibited [26]. This has led to the development of dual-blockade strategies that simultaneously target glucose and glutamine metabolism to prevent metabolic escape. Additionally, Carracedo et al. emphasize the importance of FAO as an alternative energy reservoir, particularly under nutrient stress or therapeutic pressure. These findings underscore the need for therapeutic approaches that consider the full metabolic network of OSCC rather than isolated pathways [22].

Together, these insights suggest that durable therapeutic responses in OSCC will likely require multi-target metabolic interventions, combining glycolytic inhibitors with agents that disrupt glutamine metabolism, FAO, or immune suppression. Such integrated strategies may overcome the metabolic plasticity that characterizes oral cancer cells and improve long-term treatment outcomes [5].

### 6.2. Clinical Applications and Translation Perspectives

The clinical relevance of these combinations is supported by ongoing research. For instance, the use of DCA has been evaluated in clinical trials for head and neck squamous cell carcinoma (HNSCC), including a phase II study combining DCA with cisplatin-based chemoradiotherapy in locally advanced disease (NCT01386632) [74]. Eligible patients were adults (≥18 years) with previously untreated, histologically or cytologically confirmed stage III–IVB head and neck squamous cell carcinoma, with measurable disease, adequate organ and marrow function, who were candidates for concurrent chemoradiotherapy. Key exclusions included prior HNSCC treatment or recent other malignancies, diabetes requiring hypoglycemic therapy, significant peripheral neuropathy or malabsorption, immunosuppression, allergy to compounds similar to DCA, pregnancy or breastfeeding, and concurrent anticancer therapies outside the study protocol. The addition of DCA to cisplatin-based chemoradiotherapy did not result in a significant increase in grade 3/4 adverse events, although it was associated with a higher frequency of lower-grade toxicities, particularly drug-related fever and thrombocytopenia. While DCA was associated with improved initial tumor response rates, this effect did not translate into a statistically significant improvement in survival outcomes. Although the addition of DCA to cisplatin-based chemoradiotherapy did not result in a statistically significant survival benefit, the improved initial tumor response rates provide a rationale for further investigating metabolic modulators as adjuncts to conventional chemoradiotherapy, namely in OSCC. In addition, the synergy between metabolic modulators and immunotherapy is also a growing field of interest, as it has been demonstrated that the excessive production of lactic acid in the tumor microenvironment blunts the anti-tumor activity of T and NK cells [51]. Therefore, by using agents like DCA to redirect pyruvate towards mitochondrial oxidation, it is possible to reduce lactate-mediated immunosuppression, effectively “reawakening” the immune system to attack the tumor more efficiently.

## 7. Evidence-Based Findings

**Figure 3:**
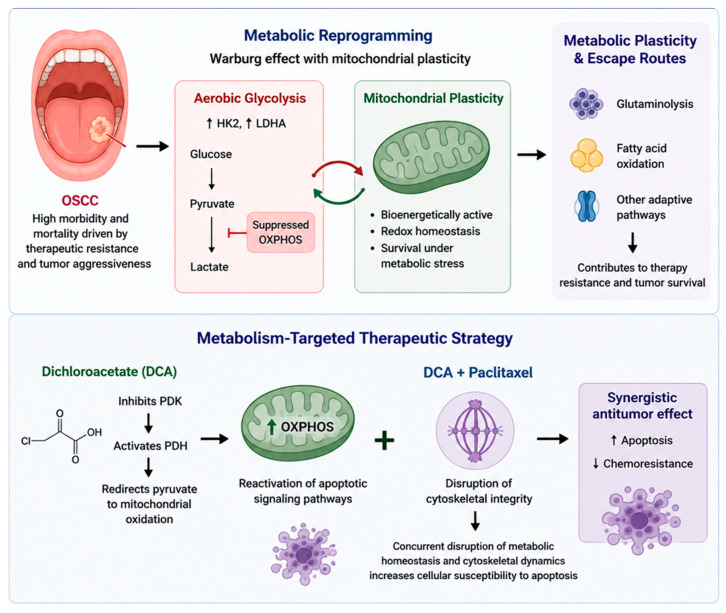
Metabolic reprogramming in oral squamous cell carcinoma (OSCC) and metabolism-targeted therapeutic strategies. The diagram illustrates the Warburg effect, characterized by increased aerobic glycolysis (↑HK2, ↑LDHA), where glucose is converted into pyruvate and subsequently lactate, alongside suppression of oxidative phosphorylation (OXPHOS). Despite this metabolic shift, tumor cells maintain mitochondrial plasticity, enabling bioenergetic activity, redox homeostasis, and survival under metabolic stress. In addition, metabolic escape routes such as glutaminolysis, fatty acid oxidation, and other adaptive pathways contribute to tumor progression, therapeutic resistance, and survival. In the lower panel, a metabolism-targeted therapeutic strategy is presented. Dichloroacetate (DCA) inhibits pyruvate dehydrogenase kinase (PDK), thereby activating pyruvate dehydrogenase (PDH) and redirecting pyruvate toward mitochondrial oxidation, restoring OXPHOS and reactivating apoptotic signaling pathways. When combined with paclitaxel, which disrupts cytoskeletal integrity, the treatment produces a synergistic antitumor effect by concurrently disturbing metabolic homeostasis and cellular structure, increasing apoptosis and reducing chemoresistance. Created by the authors using Canva Pro.

This section summarizes the main evidence identified throughout this narrative review and highlights the current strengths, and limitations regarding metabolism-targeted therapies in OSCC. Figure 3 provides an integrated overview of the metabolic reprogramming that characterizes OSCC and illustrates how inhibition of glycolysis by DCA may be bypassed through compensatory metabolic pathways, supporting the rationale for combination-based therapeutic strategies.

Recent studies indicate that OSCC is characterized by remarkable metabolic plasticity rather than exclusive dependence on aerobic glycolysis [1]. Although the Warburg effect remains a defining metabolic hallmark, mitochondria retain essential bioenergetic and biosynthetic functions that contribute to redox homeostasis, metabolic adaptation, and tumor survival [16,30,31,33]. In response to therapeutic pressure, OSCC cells can dynamically reprogram their metabolism by engaging alternative pathways, including glutaminolysis and FAO, thereby maintaining ATP production and biosynthetic capacity [22,26,45,55,56,57]. Among metabolism-targeted approaches, DCA has emerged as one of the most extensively investigated metabolic modulators. By inhibiting PDK, DCA restores PDH activity, promotes mitochondrial glucose oxidation, and partially reverses the glycolytic phenotype [10,24,33]. Furthermore, preclinical studies suggest that combining DCA with conventional chemotherapy, particularly PTX or platinum-based regimens, may enhance therapeutic efficacy through complementary metabolic and cytotoxic mechanisms [10,17,24,60,72].

Most available evidence derives from in vitro studies using OSCC cell lines and from preclinical animal models. These studies consistently demonstrate that metabolic modulation can impair tumor cell proliferation, promote apoptosis, and increase sensitivity to conventional therapies. In contrast, clinical evidence remains limited [10,17,24,33,58,59,60,72,74]. Although early-phase clinical trials in HNSCC have suggested acceptable safety profiles and improvements in initial tumor response, these findings have not translated into significant improvements in long-term survival outcomes [74]. Consequently, the current level of clinical evidence remains insufficient to support routine implementation of metabolism-targeted therapies in OSCC [63,74].

Several limitations currently hinder the clinical translation of metabolism-based therapies. First, most antitumor effects of DCA have been demonstrated using concentrations substantially higher than those safely achievable in humans [58,59,63]. Second, chronic administration remains limited by pharmacokinetic constraints and dose-dependent toxicities, particularly peripheral neuropathy [61,62,63]. In addition, DCA undergoes self-inhibition of its metabolism through GSTZ1, resulting in drug accumulation during prolonged treatment [62,63]. Perhaps most importantly, the remarkable metabolic flexibility of OSCC allows tumor cells to activate compensatory pathways, including glutaminolysis, FAO, the pentose phosphate pathway, and alternative anaplerotic pathways, thereby reducing the efficacy of single-pathway metabolic inhibition [5,22,26,33,69].

## 8. Conclusions

Current evidence supports the concept that the metabolic adaptability of OSCC represents both a hallmark of disease progression and a major obstacle to effective metabolic intervention. The coexistence of glycolytic activity with functional mitochondrial metabolism enables tumor cells to adapt to environmental stress, sustain biosynthetic demands, and develop resistance to therapeutic interventions [1]. Consequently, targeting a single metabolic pathway is unlikely to provide durable therapeutic benefit in this highly adaptable disease.

Among the metabolism-targeted strategies explored to date, DCA represents one of the most extensively investigated metabolic modulators. By inhibiting pyruvate dehydrogenase kinase, DCA promotes the restoration of mitochondrial glucose oxidation, partially reverses the glycolytic phenotype, and induces metabolic stress in highly glycolytic tumor cells. Preclinical studies consistently demonstrate reduced tumor cell viability, increased apoptosis, and enhanced sensitivity to conventional therapies. However, these encouraging findings have not yet translated into consistent clinical benefit and its role remains investigational, with clinical validation still lacking. Pharmacokinetic limitations, dose-dependent toxicities, and the ability of OSCC cells to activate compensatory metabolic pathways substantially limit the efficacy of DCA as a standalone therapeutic agent. Rather than diminishing its therapeutic relevance, these limitations reinforce the concept that metabolism-targeted therapies should be integrated into rational combination strategies. Combining DCA with chemotherapy, radiotherapy, immunotherapy, or inhibitors of complementary metabolic pathways may enhance antitumor efficacy while overcoming metabolic adaptation and therapeutic resistance.

Future research should therefore prioritize the identification and clinical validation of metabolic biomarkers capable of stratifying patient according to tumor metabolic phenotype and predicting therapeutic response. In parallel, combination-based strategies simultaneously targeting glycolysis, mitochondrial metabolism, glutaminolysis, and lipid metabolism are likely to provide greater therapeutic benefit rather than single-target approaches. Improved preclinical models that more accurately recapitulate the metabolic heterogeneity of human OSCC, together with integration of metabolic modulators like DCA with chemoradiotherapy and immunotherapy, will be essential to facilitate the clinical translation of metabolism-based therapeutic strategies. Finally, well-designed clinical trials testing DCA in combination with standard-of-care therapies in OSCC will be critical for determining its true clinical value.

## Figures and Tables

**Table 1:** Summary of the principal metabolism-targeted therapeutic strategies investigated in OSCC, highlighting their molecular targets, mechanisms of action, reported antitumor effects, translational limitations, and current stage of evidence.

Therapeutic Strategy	Molecular Target	Mechanism of Action	Principal Reported Effects in OSCC	Current Limitations	Stage of Evidence
DCA	PDK	Shifts metabolism from glycolysis to OXPHOS	Reduces cell viability; increases apoptosis and ROS production; tumor growth inhibition	Neurotoxicity; pharmacokinetic gap; autoinhibition via GSTZ1, modest efficacy as monotherapy	Preclinical (OSCC)/Limited clinical evidence
2-DG	HK2	Inhibits early glycolytic flux; induces energetic stress	Promotes mitochondrial reactivation; sensitizes cells to cisplatin	Metabolic plasticity allows bypass via other pathways	Preclinical
V-9302	ASCT2 (SLC1A5)	Inhibits the ASCT2 transporter to block the uptake of glutamine into the cell.	Targeted to suppress tumor progression and potentially abolish cancer stemness.	Compensatory metabolic flexibility (e.g., switching to FAO).	Preclinical
Glutaminase Inhibitors (e.g., CB-839)	GLS1	Blocks conversion of glutamine to glutamate	Suppresses tumor progression; drives ferroptosis; abolishes stemness	Compensatory adaptation to lipids or enhanced glycolysis	Clinical trials ongoing (Phase I/II)
FAO Inhibitors (e.g., Etomoxir)	CPT1	Blocks fatty acid oxidation; leads to ATP/NADPH depletion	Reduces invasion and lymph node metastasis; induces cell death	Significant off-target effects and hepatotoxicity	Preclinical/Limited clinical development
DCA + PTX	PDK and Cytoskeletal Microtubules	Concurrent metabolic and structural disruption	Synergistic antitumor effect; reduces chemoresistance in resistant cells	Effects largely restricted to laboratory models; requires validation	Preclinical
DCA + chemoradiotherapy	PDK and DNA-damaging mechanisms	Weakens metabolic defenses; reduces TME acidity	Improved initial tumor response rates in HNSCC patients	No significant overall survival benefit; drug-related toxicities (fever/thrombocytopenia)	Clinical evidence (Phase II study)

2-DG, 2-deoxyglucose; ATP, adenosine triphosphate; CPT1, carnitine palmitoyltransferase 1; DCA, dichloroacetate; FAO, fatty acid oxidation; GLS1, Glutaminase 1; HK2, hexokinase 2; HNSCC, head and neck squamous cell carcinoma; PDK, Pyruvate Dehydrogenase Kinase; PTX, paclitaxel; OSCC, oral squamous cell carcinoma; OXPHOS, oxidative phosphorylation; ROS, reactive oxygen species; TME, tumor microenvironment.

## Data Availability

No new data were created or analyzed in this study. Data sharing is not applicable to this article.

## Abbreviations

The following abbreviations are used in this manuscript:

2-DG	2-deoxyglucose
ATP	adenosine triphosphate
CPT1	carnitine palmitoyltransferase 1
DCA	dichloroacetate
EMT	epithelial–mesenchymal transition
FAO	fatty acid oxidation
FASN	fatty acid synthase
GLS	glutaminase
HIF-1α	hypoxia-inducible factor 1-alpha
HK2	hexokinase 2
HNSCC	head and neck squamous cell carcinoma
LDHA	lactate dehydrogenase A
MAMs	mitochondria-associated membranes
mtDNA	mitochondrial DNA
NK	natural killer
OSCC	oral squamous cell carcinoma
OXPHOS	oxidative phosphorylation
PTX	paclitaxel
PDH	Pyruvate Dehydrogenase
PDK	Pyruvate Dehydrogenase Kinase
PDK1	Pyruvate Dehydrogenase Kinase 1
PDK3	Pyruvate Dehydrogenase Kinase 3
PPP	pentose phosphate pathway
PKM2	pyruvate kinase M2
ROS	reactive oxygen species
SCD1	stearoyl-CoA desaturase
TCA	tricarboxylic acid cycle
TME	tumor microenvironment

## References

[1] Bray F., Laversanne M., Sung H., Ferlay J., Siegel R.L., Soerjomataram I., Jemal A. (2024). Global cancer statistics 2022: GLOBOCAN estimates of incidence and mortality worldwide for 36 cancers in 185 countries. CA Cancer J. Clin..

[2] Bose S., Zhang C., Le A., Le A. (2021). Glucose Metabolism in Cancer: The Warburg Effect and Beyond. The Heterogeneity of Cancer Metabolism.

[3] Hanahan D., Weinberg R.A. (2011). Hallmarks of cancer: The next generation. Cell.

[4] Vander Heiden M.G., Cantley L.C., Thompson C.B. (2009). Understanding the Warburg effect: The metabolic requirements of cell proliferation. Science.

[5] Pavlova N.N., Thompson C.B. (2016). The Emerging Hallmarks of Cancer Metabolism. Cell Metab..

[6] Zhang W., Xia M., Li J., Liu G., Sun Y., Chen X., Zhong J. (2025). Warburg effect and lactylation in cancer: Mechanisms for chemoresistance. Mol. Med..

[7] Chen J., Huang Z., Chen Y., Tian H., Chai P., Shen Y., Yao Y., Xu S., Ge S., Jia R. (2025). Lactate and lactylation in cancer. Signal Transduct. Target. Ther..

[8] Doherty J.R., Cleveland J.L. (2013). Targeting lactate metabolism for cancer therapeutics. J. Clin. Investig..

[9] Vander Heiden M.G. (2011). Targeting cancer metabolism: A therapeutic window opens. Nat. Rev. Drug Discov..

[10] Cunha A., Rocha A.C., Barbosa F., Baiao A., Silva P., Sarmento B., Queiros O. (2022). Glycolytic Inhibitors Potentiated the Activity of Paclitaxel and Their Nanoencapsulation Increased Their Delivery in a Lung Cancer Model. Pharmaceutics.

[11] Hanahan D. (2022). Hallmarks of Cancer: New Dimensions. Cancer Discov..

[12] Warburg O. (1956). On the origin of cancer cells. Science.

[13] Hsu P.P., Sabatini D.M. (2008). Cancer cell metabolism: Warburg and beyond. Cell.

[14] Huang Q., Tan Y., Yin P., Ye G., Gao P., Lu X., Wang H., Xu G. (2013). Metabolic characterization of hepatocellular carcinoma using nontargeted tissue metabolomics. Cancer Res..

[15] Semenza G.L. (2013). HIF-1 mediates metabolic responses to intratumoral hypoxia and oncogenic mutations. J. Clin. Investig..

[16] Porporato P.E., Filigheddu N., Pedro J.M.B., Kroemer G., Galluzzi L. (2018). Mitochondrial metabolism and cancer. Cell Res..

[17] Zong Y., Li H., Liao P., Chen L., Pan Y., Zheng Y., Zhang C., Liu D., Zheng M., Gao J. (2024). Mitochondrial dysfunction: Mechanisms and advances in therapy. Signal Transduct. Target. Ther..

[18] Yang L., Venneti S., Nagrath D. (2017). Glutaminolysis: A Hallmark of Cancer Metabolism. Annu. Rev. Biomed. Eng..

[19] Altman B.J., Stine Z.E., Dang C.V. (2016). From Krebs to clinic: Glutamine metabolism to cancer therapy. Nat. Rev. Cancer.

[20] Guo S., Wang X., Wang Y., Bai J., Liu Y., Shao Z. (2024). The potential therapeutic targets of glutamine metabolism in head and neck squamous cell carcinoma. Biomed. Pharmacother..

[21] Menendez J.A., Lupu R. (2007). Fatty acid synthase and the lipogenic phenotype in cancer pathogenesis. Nat. Rev. Cancer.

[22] Carracedo A., Cantley L.C., Pandolfi P.P. (2013). Cancer metabolism: Fatty acid oxidation in the limelight. Nat. Rev. Cancer.

[23] Pike L.S., Smift A.L., Croteau N.J., Ferrick D.A., Wu M. (2011). Inhibition of fatty acid oxidation by etomoxir impairs NADPH production and increases reactive oxygen species resulting in ATP depletion and cell death in human glioblastoma cells. Biochim. Biophys. Acta.

[24] Bonnet S., Archer S.L., Allalunis-Turner J., Haromy A., Beaulieu C., Thompson R., Lee C.T., Lopaschuk G.D., Puttagunta L., Bonnet S. (2007). A mitochondria-K+ channel axis is suppressed in cancer and its normalization promotes apoptosis and inhibits cancer growth. Cancer Cell.

[25] Le A., Cooper C.R., Gouw A.M., Dinavahi R., Maitra A., Deck L.M., Royer R.E., Vander Jagt D.L., Semenza G.L., Dang C.V. (2010). Inhibition of lactate dehydrogenase A induces oxidative stress and inhibits tumor progression. Proc. Natl. Acad. Sci. USA.

[26] Gross M.I., Demo S.D., Dennison J.B., Chen L., Chernov-Rogan T., Goyal B., Janes J.R., Laidig G.J., Lewis E.R., Li J. (2014). Antitumor activity of the glutaminase inhibitor CB-839 in triple-negative breast cancer. Mol. Cancer Ther..

[27] Shin J.A. (2025). Glycolytic Reprogramming in Oral Squamous Cell Carcinoma: Molecular Regulators and Natural Product-Based Therapies. Int. Dent. J..

[28] Melone M.A.B., Valentino A., Margarucci S., Galderisi U., Giordano A., Peluso G. (2018). The carnitine system and cancer metabolic plasticity. Cell Death Dis..

[29] Tufail M., Jiang C.H., Li N. (2024). Altered metabolism in cancer: Insights into energy pathways and therapeutic targets. Mol. Cancer.

[30] Zhang L., Wei Y., Yuan S., Sun L. (2023). Targeting Mitochondrial Metabolic Reprogramming as a Potential Approach for Cancer Therapy. Int. J. Mol. Sci..

[31] Rocca C., Soda T., De Francesco E.M., Fiorillo M., Moccia F., Viglietto G., Angelone T., Amodio N. (2023). Mitochondrial dysfunction at the crossroad of cardiovascular diseases and cancer. J. Transl. Med..

[32] Hsu C.C., Tseng L.M., Lee H.C. (2016). Role of mitochondrial dysfunction in cancer progression. Exp. Biol. Med..

[33] Tataranni T., Piccoli C. (2019). Dichloroacetate (DCA) and Cancer: An Overview towards Clinical Applications. Oxidative Med. Cell. Longev..

[34] Wu K., El Zowalaty A.E., Sayin V.I., Papagiannakopoulos T. (2024). The pleiotropic functions of reactive oxygen species in cancer. Nat. Cancer.

[35] Zhang C., Quinones A., Le A. (2022). Metabolic reservoir cycles in cancer. Semin. Cancer Biol..

[36] Weinberg S.E., Chandel N.S. (2015). Targeting mitochondria metabolism for cancer therapy. Nat. Chem. Biol..

[37] Tubbs E., Rieusset J. (2017). Metabolic signaling functions of ER-mitochondria contact sites: Role in metabolic diseases. J. Mol. Endocrinol..

[38] Perrone M., Caroccia N., Genovese I., Missiroli S., Modesti L., Pedriali G., Vezzani B., Vitto V.A.M., Antenori M., Lebiedzinska-Arciszewska M. (2020). The role of mitochondria-associated membranes in cellular homeostasis and diseases. Int. Rev. Cell Mol. Biol..

[39] Yang S.W., Zhang Z.G., Hao Y.X., Zhao Y.L., Qian F., Shi Y., Li P.A., Liu C.Y., Yu P.W. (2017). HIF-1alpha induces the epithelial-mesenchymal transition in gastric cancer stem cells through the Snail pathway. Oncotarget.

[40] Ferrer C.M., Lynch T.P., Sodi V.L., Falcone J.N., Schwab L.P., Peacock D.L., Vocadlo D.J., Seagroves T.N., Reginato M.J. (2014). O-GlcNAcylation regulates cancer metabolism and survival stress signaling via regulation of the HIF-1 pathway. Mol. Cell.

[41] Hong S.S., Lee H., Kim K.W. (2004). HIF-1alpha: A valid therapeutic target for tumor therapy. Cancer Res. Treat..

[42] Xie H., Hanai J., Ren J.G., Kats L., Burgess K., Bhargava P., Signoretti S., Billiard J., Duffy K.J., Grant A. (2014). Targeting lactate dehydrogenase—A inhibits tumorigenesis and tumor progression in mouse models of lung cancer and impacts tumor-initiating cells. Cell Metab..

[43] Wang Z., Zhang L., Zhang D., Sun R., Wang Q., Liu X. (2015). Glycolysis inhibitor 2-deoxy-D-glucose suppresses carcinogen-induced rat hepatocarcinogenesis by restricting cancer cell metabolism. Mol. Med. Rep..

[44] Huang Y., Sun G., Sun X., Li F., Zhao L., Zhong R., Peng Y. (2020). The Potential of Lonidamine in Combination with Chemotherapy and Physical Therapy in Cancer Treatment. Cancers.

[45] Pavlova N.N., Zhu J., Thompson C.B. (2022). The hallmarks of cancer metabolism: Still emerging. Cell Metab..

[46] Yang J., Chen F., Lang L., Yang F., Fu Z., Martinez J., Cho A., Saba N.F., Teng Y. (2024). Therapeutic Targeting of the GLS1-c-Myc Positive Feedback Loop Suppresses Glutaminolysis and Inhibits Progression of Head and Neck Cancer. Cancer Res..

[47] Shen Y.A., Chen C.L., Huang Y.H., Evans E.E., Cheng C.C., Chuang Y.J., Zhang C., Le A. (2021). Inhibition of glutaminolysis in combination with other therapies to improve cancer treatment. Curr. Opin. Chem. Biol..

[48] Wang Z., Tang S., Cai L., Wang Q., Pan D., Dong Y., Zhou H., Li J., Ji N., Zeng X. (2024). DRP1 inhibition-mediated mitochondrial elongation abolishes cancer stemness, enhances glutaminolysis, and drives ferroptosis in oral squamous cell carcinoma. Br. J. Cancer.

[49] da Silva S.D., Cunha I.W., Nishimoto I.N., Soares F.A., Carraro D.M., Kowalski L.P., Graner E. (2009). Clinicopathological significance of ubiquitin-specific protease 2a (USP2a), fatty acid synthase (FASN), and ErbB2 expression in oral squamous cell carcinomas. Oral Oncol..

[50] Aquino I.G., Bastos D.C., Cuadra-Zelaya F.J.M., Teixeira I.F., Salo T., Coletta R.D., Graner E. (2020). Anticancer properties of the fatty acid synthase inhibitor TVB-3166 on oral squamous cell carcinoma cell lines. Arch. Oral Biol..

[51] Almeida L.Y., Moreira F.D.S., Santos G., Cuadra Zelaya F.J.M., Ortiz C.A., Agostini M., Mariano F.S., Bastos D.C., Daher U.R.N., Kowalski L.P. (2023). FASN inhibition sensitizes metastatic OSCC cells to cisplatin and paclitaxel by downregulating cyclin B1. Oral Dis..

[52] Dell’Endice T.S., Posa F., Storlino G., Sanesi L., Lo Russo L., Mori G. (2025). Interplay Between Glutamine Metabolism and Other Cellular Pathways: A Promising Hub in the Treatment of HNSCC. Cells.

[53] Yoo H.C., Yu Y.C., Sung Y., Han J.M. (2020). Glutamine reliance in cell metabolism. Exp. Mol. Med..

[54] Yu W., Chen Y., Putluri N., Osman A., Coarfa C., Putluri V., Kamal A.H.M., Asmussen J.K., Katsonis P., Myers J.N. (2023). Evolution of cisplatin resistance through coordinated metabolic reprogramming of the cellular reductive state. Br. J. Cancer.

[55] Yuan L., Jiang H., Jia Y., Liao Y., Shao C., Zhou Y., Li J., Liao Y., Huang H., Pan Y. (2024). Fatty Acid Oxidation Supports Lymph Node Metastasis of Cervical Cancer via Acetyl-CoA-Mediated Stemness. Adv. Sci..

[56] Pang X., Li T.J., Shi R.J., Wan Z.X., Tang Y.Y., Tang Y.L., Liang X.H. (2024). IRF2BP2 drives lymphatic metastasis in OSCC cells by elevating mitochondrial fission-dependent fatty acid oxidation. Mol. Carcinog..

[57] Cai L., Chen Y., Tang S., Wang Q., Xu Y., Pan Y., Yang F., Chen T., Chen Q., Zhou Y. (2025). Enhanced LDL uptake and PPARalpha signaling support OSCC cell survival under glutamine deprivation. Med. Oncol..

[58] Michelakis E.D., Webster L., Mackey J.R. (2008). Dichloroacetate (DCA) as a potential metabolic-targeting therapy for cancer. Br. J. Cancer.

[59] Stacpoole P.W., Henderson G.N., Yan Z., James M.O. (1998). Clinical pharmacology and toxicology of dichloroacetate. Environ. Health Perspect..

[60] Xie Q., Zhang H.F., Guo Y.Z., Wang P.Y., Liu Z.S., Gao H.D., Xie W.L. (2015). Combination of Taxol(R) and dichloroacetate results in synergistically inhibitory effects on Taxol-resistant oral cancer cells under hypoxia. Mol. Med. Rep..

[61] Stacpoole P.W., Martyniuk C.J., James M.O., Calcutt N.A. (2019). Dichloroacetate-induced peripheral neuropathy. Int. Rev. Neurobiol..

[62] Bianchi C., Martinelli R.P., Rozados V.R., Scharovsky O.G. (2024). Use of sodium dichloroacetate for cancer treatment: A scoping review. Medicina.

[63] Koltai T., Fliegel L. (2024). Dichloroacetate for Cancer Treatment: Some Facts and Many Doubts. Pharmaceuticals.

[64] Nguyen T.Q., Phan U.T.T., Can M.V., Nguyen D.H., Han B., Hoang B.X. (2025). Synergistic anti-tumor effect of fenbendazole and diisopropylamine dichloroacetate in immunodeficient BALB/c nude mice transplanted with A549 lung cancer cells. Transl. Lung Cancer Res..

[65] She W., Liu T., Li H., Wang Z., Guo Z., Liu Y., Liu Y. (2023). Reprogramming Energy Metabolism with Synthesized PDK Inhibitors Based on Dichloroacetate Derivatives and Targeted Delivery Systems for Enhanced Cancer Therapy. J. Med. Chem..

[66] Rostamian H., Khakpoor-Koosheh M., Jafarzadeh L., Masoumi E., Fallah-Mehrjardi K., Tavassolifar M.J., Pawelek J.M., Mirzaei H.R., Hadjati J. (2022). Restricting tumor lactic acid metabolism using dichloroacetate improves T cell functions. BMC Cancer.

[67] Leone R.D., Powell J.D. (2020). Metabolism of immune cells in cancer. Nat. Rev. Cancer.

[68] Al-Azawi A., Sulaiman S., Arafat K., Yasin J., Nemmar A., Attoub S. (2021). Impact of Sodium Dichloroacetate Alone and in Combination Therapies on Lung Tumor Growth and Metastasis. Int. J. Mol. Sci..

[69] Martinez-Outschoorn U.E., Peiris-Pages M., Pestell R.G., Sotgia F., Lisanti M.P. (2017). Cancer metabolism: A therapeutic perspective. Nat. Rev. Clin. Oncol..

[70] Ruggieri V., Agriesti F., Scrima R., Laurenzana I., Perrone D., Tataranni T., Mazzoccoli C., Lo Muzio L., Capitanio N., Piccoli C. (2015). Dichloroacetate, a selective mitochondria-targeting drug for oral squamous cell carcinoma: A metabolic perspective of treatment. Oncotarget.

[71] Haugrud A.B., Zhuang Y., Coppock J.D., Miskimins W.K. (2014). Dichloroacetate enhances apoptotic cell death via oxidative damage and attenuates lactate production in metformin-treated breast cancer cells. Breast Cancer Res. Treat..

[72] Simons A.L., Ahmad I.M., Mattson D.M., Dornfeld K.J., Spitz D.R. (2007). 2-Deoxy-D-glucose combined with cisplatin enhances cytotoxicity via metabolic oxidative stress in human head and neck cancer cells. Cancer Res..

[73] Lin J., Fang W., Xiang Z., Wang Q., Cheng H., Chen S., Fang J., Liu J., Wang Q., Lu Z. (2023). Glycolytic enzyme HK2 promotes PD-L1 expression and breast cancer cell immune evasion. Front. Immunol..

[74] Powell S.F., Mazurczak M., Dib E.G., Bleeker J.S., Geeraerts L.H., Tinguely M., Lohr M.M., McGraw S.C., Jensen A.W., Ellison C.A. (2022). Phase II study of dichloroacetate, an inhibitor of pyruvate dehydrogenase, in combination with chemoradiotherapy for unresected, locally advanced head and neck squamous cell carcinoma. Investig. New Drugs.

